# When Current Severity Overrides Reassuring Findings: TG18‐Guided Biliary Drainage in ERCP‐Confirmed Purulent Cholangitis

**DOI:** 10.1002/ccr3.73674

**Published:** 2026-10-02

**Authors:** Hatem Mousa Taha, Khaled Hatem Taha

**Affiliations:** ^1^ Department of Internal Medicine Palestine Medical Complex Ramallah Palestine; ^2^ Faculty of Medicine and Health Sciences An‐Najah National University Nablus Palestine

**Keywords:** acute cholangitis, biliary drainage, biliary sludge, cholecystectomy, ERCP, Tokyo guidelines 2018

## Abstract

A 42‐year‐old antibiotic‐naïve man with previous laparoscopic cholecystectomy and poorly controlled type 2 diabetes mellitus (HbA1c 9.7%) presented with Charcot's triad 7 days after a formally documented normal hepatobiliary evaluation (alanine aminotransferase [ALT] 22 U/L; total bilirubin 0.7 mg/dL [12 μmol/L]; common bile duct [CBD] ≤ 5 mm on ultrasound). Admission findings satisfied all three Tokyo Guidelines 2018 (TG18) diagnostic domains. Severity was Grade II on two of the five TG18 severity criteria—leukocytosis (14.2 × 10^3^/μL) and hyperbilirubinaemia (26 mg/dL, 445 μmol/L); temperature (38.9°C), age (42 years), and albumin (3.4 g/dL, 34 g/L) did not meet their thresholds, and no Grade III organ dysfunction was identified on the recorded variables. Grade II severity established the indication for early biliary drainage at presentation. Computed tomography (CT) showed only mild intrahepatic ductal dilatation with 3–4 hyperattenuating distal CBD filling defects (greatest ≈4 mm) and no dominant calculus. Immediate endoscopic retrograde cholangiopancreatography (ERCP) was not feasible because of endoscopy‐suite and anesthetic availability; antimicrobial and supportive therapy continued, and persistent clinical and biochemical abnormalities at 48 h (ALT 837 U/L; bilirubin 24 mg/dL, 410 μmol/L; C‐reactive protein 86 mg/L) reinforced the urgency of drainage during that interval. ERCP on Day 3, at the earliest available opportunity, showed macroscopic purulent bile at ductal entry, distal CBD filling defects, and copious biliary sludge on balloon sweep. Fever resolved within 24 h, and hepatobiliary and inflammatory markers normalized by 3 months (ALT 38 U/L; bilirubin 0.9 mg/dL, 15 μmol/L; C‐reactive protein < 5 mg/L). This case illustrates that current severity, not antecedent normality or unimpressive imaging, should govern the timing of biliary drainage, and it reports transparently an interval to drainage imposed by resource availability.

## Key Clinical Message

In acute cholangitis, current TG18 severity—not a recently normal hepatobiliary evaluation or unimpressive imaging—determines management. Grade II severity at presentation establishes the indication for early biliary drainage alongside antimicrobial and supportive therapy.

## 1 Introduction

The Tokyo Guidelines 2018 (TG18) set the urgency of biliary drainage in acute cholangitis by the patient's current severity grade rather than by the magnitude of the biochemical derangement or the conspicuousness of imaging [1, 2, 3]. Grade II (moderate) disease establishes an indication for early biliary drainage at presentation, alongside antimicrobial and supportive therapy [1, 2]. That principle matters most when imaging is unimpressive and when the most recent hepatobiliary investigations were normal. Prior reassuring information can contribute to cognitive bias in clinical decision‐making [4], which is one reason TG18 requires severity to be graded prospectively and reassessed during admission [1, 5].

We report a case in which Grade II severity was identified at presentation and drainage was indicated from that point, but in which immediate endoscopy was unavailable. The contribution is a documented trajectory: verified normality 7 days earlier, TG18‐based diagnostic and severity assessment, a transparently reported resource‐imposed interval to drainage, ERCP‐proven purulent cholangitis and normalization of hepatobiliary and inflammatory markers by 3 months. The report follows the CARE reporting guidelines.

## 2 Case History and Examination

### 2.1 Documented Normal Baseline—Day −7 (09 February 2026)

A 42‐year‐old Palestinian man attended a routine outpatient gastroenterology review on 09 February 2026. His history comprised laparoscopic cholecystectomy 2 years earlier (clips later confirmed on imaging in expected position, without migration or Mirizzi‐type morphology) and poorly controlled type 2 diabetes mellitus (T2DM; HbA1c 9.7%; metformin 1000 mg twice daily). He took no other regular medication, denied alcohol use, and had no known liver disease. Abdominal ultrasound showed a CBD of ≤ 5 mm with no calculus or dilatation, hepatomegaly with moderate fatty infiltration, and an incidental splenic lesion unrelated to the biliary tree. Liver biochemistry, platelets, and renal function were normal (Table 1, Day −7). Both the biochemistry and the biliary imaging were therefore formally documented as normal 7 days before presentation.

**TABLE 1:** Serial biochemical parameters, with local reference intervals, from the documented normal baseline (Day −7) through admission, the 48‐h reassessment, ERCP (Day 3), and three‐month follow‐up. All dates February–May 2026.

Parameter	Reference interval	Day −7 baseline	Day 0 admission	Day 2 pre‐ERCP	Day 5 post‐ERCP	Day 7–8	Month 3
ALT (U/L)	0–55	22	1020^a^	837^a^	521^a^	189^a^	38
AST (U/L)	5–34	18	564^a^	384^a^	210^a^	74^a^	29
GGT (U/L)	12–64	31	312^a^	278^a^	180^a^	89^a^	41
ALP (U/L)	40–150	88	298^a^	261^a^	194^a^	130	102
Total bilirubin (mg/dL)	0.2–1.2	0.7	26^a^	24^a^	14^a^	2.1^a^	0.9
Direct bilirubin (mg/dL)	0.0–0.5	0.2	18^a^	17^a^	9^a^	1.1^a^	0.3
Albumin (g/dL)	3.5–5.0	4.2	3.4^b^	3.3^b^	3.6	3.9	4.1
INR	Not issued^c^	1.0	1.3	1.2	1.1	1.0	1.0
Platelets (×10^3^/μL)	150–400	245	228	215	236	252	268
Amylase (U/L)	25–125	NM	4830^a^	501^a^	142^a^	88	NM
Lipase (U/L)	8–78	NM	680^a^	420^a^	190^a^	95^a^	NM
CRP (mg/L)	< 5	NM	> 100^a^	86^a^	38^a^	9^a^	< 5
WBC (×10^3^/μL)	4.0–11.0	NM	14.2^a^	12.8^a^	9.4	7.2	6.8
Fasting glucose (mg/dL)	70–100	182^a^	214^a^	196^a^	138^a^	112^a^	97
HbA1c (%)	Not applicable	9.7^d^	—	—	—	—	8.3^e^
Creatinine (mg/dL)	0.7–1.3	0.9	1.1	1.0	0.9	0.9	0.9

*Note:* The symbols a and b denote deviation from the laboratory reference interval only; they do not indicate that a value satisfies any TG18 severity criterion. SI conversion: total and direct bilirubin (mg/dL) × 17.1 = μmol/L; fasting glucose (mg/dL) × 0.0555 = mmol/L; creatinine (mg/dL) × 88.4 = μmol/L; albumin (g/dL) × 10 = g/L. Counts expressed as ×10^3^/μL are numerically identical to ×10^9^/L. Reference intervals are those of the clinical laboratory at Palestine Medical Complex, Ramallah. ERCP was performed on Day 3 (19 February 2026); the “Day 2 (Pre‐ERCP)” column reports the 48‐h reassessment sample drawn on 18 February 2026.

Abbreviations: ALP, alkaline phosphatase; ALT, alanine aminotransferase; AST, aspartate aminotransferase; CRP, C‐reactive protein; ERCP, endoscopic retrograde cholangiopancreatography; GGT, gamma‐glutamyl transferase; HbA1c, glycated hemoglobin A1c; INR, international normalized ratio; NM, not measured; WBC, white blood cell count.

^a^ Above the upper reference limit.

^b^ Below the lower reference limit.

^c^ A separate reference interval for INR is not issued by the reporting laboratory; INR is interpreted here only against the TG18 Grade III coagulation threshold (> 1.5), which was not reached.

^d^ HbA1c at baseline (Day −7).

^e^ HbA1c at three‐month follow‐up.

### 2.2 Interval Endoscopy—Day −4 (12 February 2026)

Four days later an elective ileocolonoscopy was performed for chronic ileocaecal changes. It showed three small terminal‐ileal ulcers with normal intervening mucosa; biopsies were obtained and a rectal polyp was removed. Histology was still pending at the time of the acute admission and is reported in Section 4. Concurrent upper gastrointestinal endoscopy showed a positive rapid urease test. **There was no biliary instrumentation, no sphincterotomy**, **and no antibiotic prophylaxis**, so the procedure is not a plausible cause of the biliary event that followed.

### 2.3 Acute Presentation—Day 0 (16 February 2026)

He presented with a 2‐day history of fever, progressive right upper quadrant and epigastric pain, nausea and jaundice. Examination: temperature 38.9°C; pulse 108/min; blood pressure 118/74 mmHg (mean arterial pressure [MAP] 89 mmHg); SpO_2_ 98% on room air; Glasgow Coma Scale (GCS) 15; scleral icterus; right upper quadrant and epigastric tenderness. Murphy's sign was not elicitable post‐cholecystectomy. Charcot's triad was complete. He had received no antimicrobial therapy before admission, which matters for the culture results below.

### 2.4 TG18 Diagnostic Criteria and Severity Grading

#### 2.4.1 Diagnosis

All three TG18 diagnostic domains were satisfied, giving a *definite* diagnosis of acute cholangitis: [1] **(A)** fever 38.9°C with leukocytosis 14.2 × 10^3^ /μL and C‐reactive protein (CRP) > 100 mg/L; **(B)** jaundice with total bilirubin 26 mg/dL (445 μmol/L) and abnormal liver biochemistry (Table 1); **(C)** mild intrahepatic biliary dilatation with hyperattenuating dependent material in the distal CBD indicating an etiology.

#### 2.4.2 Severity Grade

Severity was graded at diagnosis and reassessed at 24 and 48 h [1, 5]. Two of the five Grade II criteria were met: **leukocytosis** (14.2 × 10^3^/μL; criterion > 12 or < 4 × 10^3^/μL) and **hyperbilirubinaemia** (26 mg/dL, 445 μmol/L; criterion ≥ 5 mg/dL). Three were not: temperature 38.9°C (criterion ≥ 39°C); age 42 years (criterion ≥ 75); and albumin 3.4 g/dL (34 g/L), above the hypoalbuminaemia threshold of 2.45 g/dL (24.5 g/L; 0.7 × the local lower reference limit of 3.5 g/dL). Any two are sufficient, so the patient was **Grade II (moderate)**. Charcot's triad—complete here—is a diagnostic construct and confers no severity grade.

**Grade II severity established the indication for early biliary drainage at presentation**, alongside antimicrobial and supportive therapy [1, 2].

No Grade III organ dysfunction was identified on the recorded variables: [1] *cardiovascular*—118/74 mmHg (MAP 89 mmHg), no vasopressor; *neurological*—GCS 15; *renal*—creatinine 1.1 mg/dL (97 μmol/L), below the > 2.0 mg/dL (177 μmol/L) threshold; *hepatic*—INR 1.3, below > 1.5; *hematological*—platelets 228 × 10^3^/μL, above the < 100 × 10^3^/μL threshold. Two components could not be formally assessed: the respiratory criterion (PaO_2_/FiO_2_ < 300), because no arterial blood gas was performed and only SpO_2_ (98% on room air, no oxygen requirement) is recorded; and the oliguria component, because quantitative urine output is unavailable. Both appear in the Limitations.

## 3 Investigations, Differential Diagnosis and Treatment

### 3.1 Investigations

Admission and serial biochemistry, with local reference intervals, are shown in Table 1. ALT was 1020 U/L on admission—46‐fold the Day −7 baseline and 18.5 times the upper reference limit of 55 U/L—with a mixed hepatocellular and cholestatic pattern (aspartate aminotransferase 564, gamma‐glutamyl transferase 312, alkaline phosphatase 298 U/L).

Aminotransferase elevation of this magnitude is often taken to indicate hepatocellular rather than biliary disease. Marked elevations above 1000 U/L have, however, been reported in acute choledocholithiasis—pooled frequency 7.8% (95% CI 5.5–10.8) [6] and earlier case series [7]—and are therefore compatible with an acute biliary obstructive presentation. Two features favored biliary obstruction over ischaemic (hypoxic) hepatopathy: no hypotensive episode was documented, MAP remaining at 89 mmHg or above; and the trajectory was a progressive stepwise decline (ALT −81% over 7 days) rather than the massive but transient rise characteristic of hypoxic hepatitis [8]. The temporal improvement following ERCP, while antimicrobial and supportive therapy continued, favored a biliary mechanism but does not establish causality; hypoxic hepatopathy is not formally excluded (Limitations).

Pancreatic enzymes were markedly elevated on admission (amylase 4830 U/L, 38.6 times the upper reference limit of 125 U/L; lipase 680 U/L) and fell by 97% and 72% respectively by Day 5. Hyperamylasaemia without clinical pancreatitis has been described in acute biliary disease [9]. However, epigastric pain with amylase above three times the upper reference limit satisfies two of the three Revised Atlanta criteria, so concurrent mild biliary pancreatitis cannot be excluded [10]. A normal pancreatic appearance on CT does not exclude early pancreatitis, and the fall in enzymes after ERCP does not itself prove an obstructive mechanism. The distinction did not alter management.

#### 3.1.1 Cross‐Sectional Imaging

Triphasic CT showed mild intrahepatic ductal dilatation; hyperattenuating dependent material in the distal CBD (3–4 filling defects, greatest ≈4 mm); mild periductal fat stranding; no dominant calculus; no pancreatic or peripancreatic change; and clips in expected position. Two sets of blood cultures were drawn before the first antibiotic dose (Figure 1A).

**FIGURE 1:**
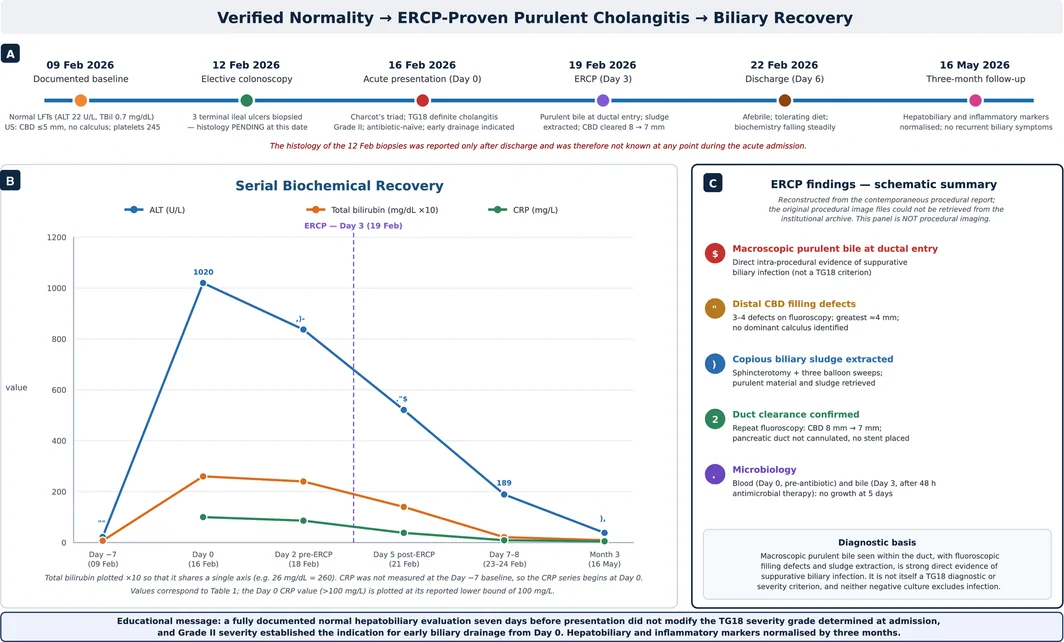
Clinical timeline and biochemical recovery. (A) Timeline from the documented normal hepatobiliary baseline (09 February 2026), through colonoscopy with terminal ileal biopsy (12 February 2026; histology pending at that date), acute presentation with TG18 Grade II cholangitis (16 February 2026), ERCP (19 February 2026) and discharge (22 February 2026), to three‐month follow‐up (16 May 2026). (B) Serial curves for ALT (U/L), total bilirubin (mg/dL, plotted ×10 to share a single axis) and CRP (mg/L); CRP was not measured at the Day −7 baseline, so that series begins at Day 0, and the Day 0 CRP value, reported as > 100 mg/L, is plotted at its lower bound of 100 mg/L. (C) Schematic summary of the ERCP findings, reconstructed from the contemporaneous procedural report. The original procedural fluoroscopic and endoscopic image files could not be retrieved from the institutional archive. (C) is a schematic and is not a reproduction of procedural imaging.

### 3.2 Differential Diagnosis

**TG18 Grade II acute cholangitis due to distal CBD sludge obstruction—final diagnosis**, supported at ERCP by macroscopic purulent bile at ductal entry, fluoroscopic filling defects, and the extraction of copious sludge (Figure 1C).

#### 3.2.1 Choledocholithiasis

No dominant calculus was seen on CT or ERCP fluoroscopy. This does not exclude choledocholithiasis: microlithiasis within the extracted material, or a small stone that had already passed, remains possible, and the aspirate was not analyzed. Biliary sludge and microlithiasis are closely related entities that may represent different stages of the same process, and they are not separated by imaging alone [11, 12].

#### 3.2.2 Clip‐Related Biliary Complication

Clip‐related biliary complication (migration into the duct; Mirizzi‐type compression). Not supported: CT showed clips in expected position without compressive morphology.

#### 3.2.3 Acute Viral or Drug‐Induced Hepatitis

Considered given an ALT of 1020 U/L, and not supported: hepatitis B surface antigen, anti‐hepatitis C virus antibody, and anti‐hepatitis A virus IgM were negative; medication review identified no new drug, herbal remedy, or supplement, and alcohol use was denied. Hepatitis E serology was not performed (Limitations).

#### 3.2.4 Post‐ERCP Pancreatitis

Not supported: the enzyme rise preceded ERCP by 3 days and the pancreatic duct was not cannulated.

### 3.3 Treatment and Course to Drainage

Management followed the TG18 Grade II pathway (Figure 2) [2]. Intravenous piperacillin–tazobactam 4.5 g every 8 h (q8h; 13.5 g/day) was started immediately after blood cultures were drawn, consistent with TG18 antimicrobial recommendations [13], with fluid resuscitation, fasting, analgesia and basal–bolus insulin (glargine 18 U nocte; lispro 4 U three times daily pre‐meal; metformin withheld). This was the first antimicrobial exposure of the illness.

**FIGURE 2:**
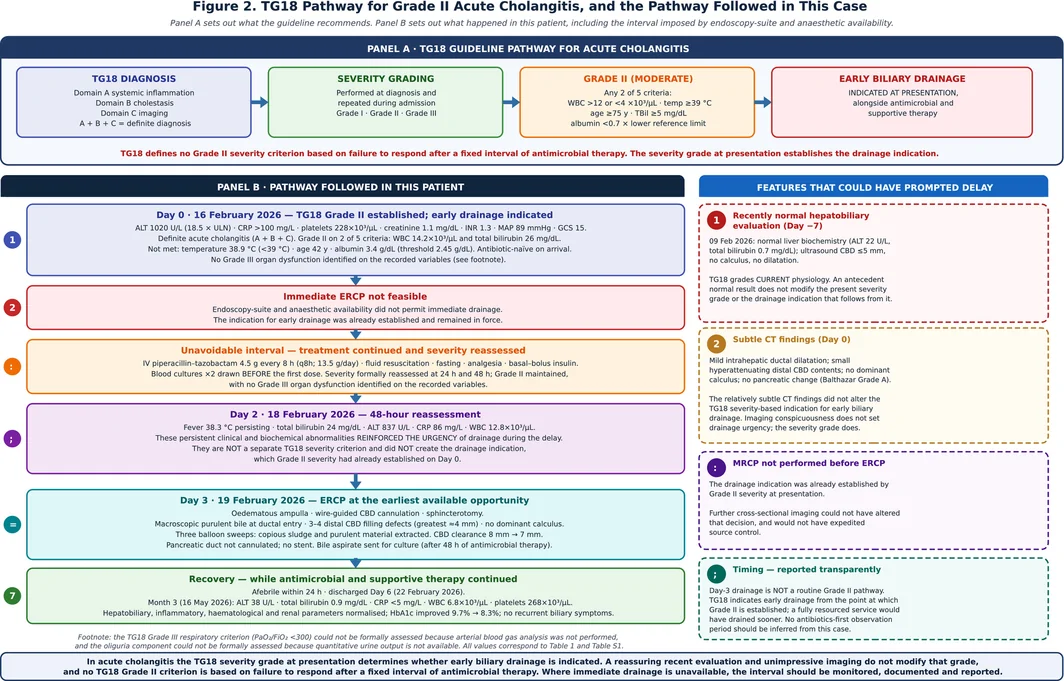
TG18 pathway for Grade II acute cholangitis and the pathway followed in this case. (A) Shows the guideline pathway: TG18 diagnosis (domains A + B + C), severity grading, and—for Grade II—early biliary drainage indicated at presentation alongside antimicrobial and supportive therapy. (B) Shows what happened in this patient: Grade II established on Day 0 with early drainage indicated; immediate ERCP not feasible because of endoscopy‐suite and anesthetic availability; antimicrobial and supportive therapy continued during the unavoidable interval with severity reassessed at 24 and 48 h; persistent clinical and biochemical abnormalities at 48 h reinforcing the urgency of drainage without constituting a separate severity criterion; ERCP on Day 3 at the earliest available opportunity with the documented procedural findings; and subsequent improvement while antimicrobial and supportive therapy continued. Annotations on the right record three features that could have prompted delay and the reason each did not, together with a transparency note on timing. All numerical values correspond to Table 1 and Table S1.

Grade II severity had established an indication for early biliary drainage from the day of admission. Immediate ERCP was not feasible because of endoscopy‐suite and anesthetic availability. Throughout that interval the patient remained hemodynamically stable, with no Grade III organ dysfunction on the recorded variables, while antimicrobial and supportive therapy continued and severity was reassessed at 24 and 48 h.

**Day 1 (17 February 2026)**. Fever persisted (38.5°C) with unchanged jaundice and tenderness; severity remained Grade II.

**Day 2 (18 February 2026)—48‐h reassessment**. Fever persisted (38.3°C), jaundice persisted (total bilirubin 24 mg/dL, 410 μmol/L) and the biochemical response was inadequate (ALT 837 U/L; CRP 86 mg/L; white cell count 12.8 × 10^3^/μL). These persistent abnormalities reinforced the urgency of drainage during the unavoidable delay; they did not constitute a separate TG18 severity criterion and did not create the drainage indication, which Grade II severity had already established at presentation. Magnetic resonance cholangiopancreatography was not performed: the indication was already established and further imaging could not have altered it [2, 3, 14].

### 3.4 ERCP—Day 3 (19 February 2026)

ERCP was performed on Day 3 at the earliest available opportunity. The ampulla was oedematous; wire‐guided CBD cannulation and sphincterotomy were performed. On aspiration, macroscopic purulent bile was identified at ductal entry—direct intra‐procedural evidence of suppurative biliary infection, though not itself a TG18 criterion. Fluoroscopy showed 3–4 distal CBD filling defects (greatest ≈4 mm). Three balloon sweeps extracted copious sludge and purulent material; repeat fluoroscopy confirmed clearance (CBD 8 mm before, 7 mm after). The pancreatic duct was not cannulated and no stent was placed. Bile aspirate was sent for culture.

#### 3.4.1 Microbiology

The admission blood cultures showed no growth at 5 days, and the bile culture subsequently showed no growth at 5 days. The two results require different explanations. The blood cultures were drawn before any antimicrobial was given: bacteraemia is documented in only a proportion of patients with acute cholangitis [13, 15]. The bile aspirate was obtained after 72 h of piperacillin–tazobactam, and preceding antimicrobial exposure may have reduced culture yield [16]. Neither result excludes biliary infection.

## 4 Outcome and Follow‐Up

Recovery was prompt (Figure 1B). Fever resolved within 24 h of ERCP and tenderness diminished. By Day 5, total bilirubin had fallen to 14 mg/dL (239 μmol/L) and amylase to 142 U/L; by Days 7–8, ALT was 189 U/L, bilirubin 2.1 mg/dL (36 μmol/L) and CRP 9 mg/L. He was discharged on Day 6 (22 February 2026), afebrile and tolerating a normal diet.

At three‐month follow‐up (16 May 2026), hepatobiliary, inflammatory, hematological, and renal parameters had normalized: ALT 38 U/L; bilirubin 0.9 mg/dL (15 μmol/L); CRP < 5 mg/L; white cell count 6.8 × 10^3^/μL; platelets 268 × 10^3^/μL; creatinine 0.9 mg/dL (80 μmol/L); fasting glucose 97 mg/dL (5.4 mmol/L). HbA1c improved from 9.7% to 8.3% and therefore remained abnormal. There had been no recurrent biliary symptoms.

The terminal‐ileal biopsy was subsequently reported as **mild active chronic ileitis**, characterized histologically as nonspecific. This finding did not inform the acute biliary management and no causal role in the biliary event is claimed.

### 4.1 Final Diagnosis

ERCP‐supported TG18 Grade II purulent cholangitis due to distal CBD sludge obstruction after cholecystectomy.

## 5 Discussion

### 5.1 Current Severity as the Management Driver

The recently normal evaluation is not the teaching point; it is the setting that makes it visible, which is that current TG18 severity supersedes it. Grade II severity was established at presentation on objective criteria and, with it, the indication for early biliary drainage [1, 2]. A normal evaluation 7 days earlier, and only mildly abnormal CT, could each have supplied reassurance when evolving severity should instead have driven escalation—neither did so here.

### 5.2 Why the Grade Was II, and Why the Distinction Matters

We set out the grading arithmetic explicitly (Section 2.4) because it is a common source of error. Charcot's triad establishes suspicion and contributes to the diagnostic domains but is not a severity instrument: a patient may have a complete triad and remain Grade I. Conversely, this patient met Grade II on leukocytosis and hyperbilirubinaemia alone. Equally, TG18 defines no Grade II severity criterion based on failure to respond after a fixed interval of antimicrobial therapy; the grade at presentation, not a later response assessment, established the drainage indication here.

### 5.3 Imaging Findings and the Decision Not to Perform MRCP

The relatively subtle CT findings did not alter the TG18 severity‐based indication for early biliary drainage. More generally, initial ultrasound has been reported to outperform initial CT in patients suspected of having acute biliary disease [17]. We make no claim here about the sensitivity of CT for any particular type of ductal content. MRCP was not performed because the drainage indication was already established and further imaging could not have altered it or expedited source control [2, 3, 14].

### 5.4 Timing of Drainage, Reported Transparently

TG18 Grade II severity established the indication for early biliary drainage at presentation. Persistent abnormalities at 48 h reinforced the urgency of drainage during the unavoidable delay; they did not constitute a separate TG18 severity criterion or create the initial indication. Immediate ERCP was not feasible because of endoscopy‐suite and anesthetic availability; the patient remained hemodynamically stable, with no Grade III organ dysfunction on the recorded variables, while treatment continued. ERCP followed on Day 3 at the earliest available opportunity.

We report this rather than presenting it as ideal. Drainage took place approximately 72 h after admission, beyond the 48‐h window suggested by the ASGE for biliary decompression in cholangitis [14]. Day‐3 drainage is therefore not a model Grade II pathway, and a fully resourced service would have drained sooner. Where immediate drainage is unavailable, scheduled reassessment keeps such an interval monitored and documented rather than unexamined. The favorable outcome does not validate delay, and no antibiotics‐first observation period should be inferred.

### 5.5 Biliary Sludge After Cholecystectomy

Despite previous cholecystectomy, ERCP demonstrated obstructing ductal sludge in this patient. We do not offer a mechanistic explanation for that finding. The composition of the extracted material was not analyzed, no bile‐acid or lipid measurement is available, and we are not able to attribute the event to any particular predisposing factor. Reporting the observation without a mechanism is the accurate position here.

### 5.6 Interpreting the Negative Cultures

The two negative cultures illustrate a distinction that is easily collapsed. Sterile blood cultures drawn before any antimicrobial exposure need no special explanation, since bacteraemia is documented in only a proportion of patients with acute cholangitis [13, 15]. For bile obtained after approximately 72 h of antimicrobial therapy, that exposure may have reduced culture yield [16]. Neither excludes biliary infection, and neither indicates that antibiotics necessarily sterilize bile. Bile sampling during cholangiography may aid microbiological characterization and antimicrobial tailoring [16]; this concerns what is done during a procedure and does not justify delaying antimicrobial therapy in acute cholangitis.

### 5.7 Limitations

**First**, no organism was identified; the aetiological attribution rests on intra‐procedural and fluoroscopic findings rather than microbiology. **Second**, hypoxic hepatopathy is not formally excluded. **Third**, concurrent mild biliary pancreatitis cannot be excluded: two of three Revised Atlanta criteria were satisfied and a normal pancreatic CT does not exclude early pancreatitis [10]. **Fourth**, the Grade III respiratory criterion could not be formally assessed (no arterial blood gas), nor the oliguria component (quantitative urine output unavailable). **Fifth**, hepatitis E serology was not performed. **Sixth**, the extracted material was not analyzed, so microlithiasis and a passed stone are not excluded. **Seventh**, a documented archive search did not retrieve the original ERCP image files; the findings are transcribed from the contemporaneous report and Figure 1C is a schematic. **Eighth**, no causal role in the biliary event is claimed for the incidental ileal finding reported in Section 4. **Ninth**, drainage was on Day 3 rather than earlier (Section 5.4). **Tenth**, this is a single case report: no causal inference or generalization is warranted.

## 6 Clinical Pearls

- A recently normal hepatobiliary evaluation does not exclude acute cholangitis developing days later; current severity, not antecedent normality, must govern management.
- TG18 Grade II requires any two of: white cell count > 12 or < 4 × 10^3^/μL; temperature ≥ 39°C; age ≥ 75 years; bilirubin ≥ 5 mg/dL; albumin < 0.7 × the lower reference limit. Charcot's triad confers no severity grade.
- Grade II severity at presentation is itself the indication for early biliary drainage. TG18 defines no criterion by which failure to respond after a fixed interval creates that indication, and no routine antibiotics‐first observation period should be inferred.
- Relatively subtle cross‐sectional imaging does not exclude purulent biliary infection, and it does not modify the severity grade on which the drainage indication rests.
- Marked aminotransferase elevation above 1000 U/L is compatible with acute biliary obstruction and should not by itself divert the diagnostic pathway towards primary hepatocellular disease.
- Negative blood cultures drawn before antibiotics and negative bile cultures drawn afterwards mean different things, and neither excludes biliary infection. Bile sampling during cholangiography may assist microbiological characterization, but antimicrobial therapy should never be delayed to obtain it.

## Author Contributions

**Hatem Mousa Taha:** conceptualization, investigation, data curation, visualization, writing – original draft, writing – review and editing, supervision. **Khaled Hatem Taha:** investigation, writing – review and editing.

## Funding

The authors have nothing to report.

## Disclosure

*Image Availability*: No imaging or histopathology material accompanies this report. A documented search of the institutional picture archiving and communication system and of the endoscopy unit records was undertaken for this revision; the original ERCP fluoroscopic and endoscopic image files could not be retrieved. The admission CT images and the terminal ileal histopathology slides are likewise not available or retrievable for submission. The procedural findings reported here are transcribed from the contemporaneous procedural report, and Figure 1C is a schematic summary of that report and not a reproduction of procedural imaging. The authors make no undertaking to supply imaging or histopathology material.

## Ethics Statement

This work was conducted in accordance with the Declaration of Helsinki. Under the institutional policy applicable at Palestine Medical Complex, Ramallah, formal research ethics committee approval was not required for this single, anonymised case report. No ethics waiver reference number was issued.

## Consent

Written informed consent for publication of this case report was obtained from the patient before manuscript submission, in accordance with Wiley's patient consent policy. A copy of the signed consent form is available to the journal on request.

## Conflicts of Interest

The authors declare no conflicts of interest.

## Supporting information


**Table S1:** Detailed chronological clinical timeline from the documented normal baseline (Day −7, 09 February 2026) to three‐month follow‐up (16 May 2026).

## Data Availability

The data that support the findings of this study are available on request from the corresponding author. The data are not publicly available due to privacy or ethical restrictions.
