# Supplementary material for: When Current Severity Overrides Reassuring Findings: TG18‐Guided Biliary Drainage in ERCP‐Confirmed Purulent Cholangitis
Source: Clin Case Rep. 2026 Oct 2;14(10):e73674. doi: 10.1002/ccr3.73674 (PMC13633413; doi:10.1002/ccr3.73674)
Supplement: Supplementary file 1 [file CCR3-14-e73674-s001.docx]

*Supporting Information · File S1 · Taha & Taha · Clinical Case Reports · MS 5071002*

**Table S1. Detailed chronological clinical timeline**

Chronological record from the documented normal hepatobiliary baseline (Day −7, 09 February 2026) to three-month follow-up (16 May 2026). Every value corresponds to Table 1 of the main manuscript, to Figure 1 and to Figure 2. Entries are restricted to what the source clinical record documents. Where a variable was not measured, or where a treatment or follow-up detail is not established by the source record, no entry is made; nothing is inferred or reconstructed.

| **Date** | **Day** | **Event** | **Clinical details and biochemistry** | **Actions and documented results** |
| --- | --- | --- | --- | --- |
| 09 Feb 2026 | Day −7 | **Outpatient gastroenterological evaluation — documented normal baseline** | Ultrasound: CBD ≤5 mm; no calculus; no biliary dilatation; hepatomegaly with moderate fatty infiltration; incidental splenic hyperechoic lesion, unrelated to the biliary tree.  Biochemistry normal: ALT 22; AST 18; GGT 31; ALP 88 U/L; total bilirubin 0.7 mg/dL (12 µmol/L); direct bilirubin 0.2 mg/dL; albumin 4.2 g/dL (42 g/L); INR 1.0; platelets 245 × 10³/µL; creatinine 0.9 mg/dL (80 µmol/L). HbA1c 9.7%; fasting glucose 182 mg/dL (10.1 mmol/L). | No biliary intervention indicated. Metformin 1000 mg twice daily continued. |
| 12 Feb 2026 | Day −4 | **Elective colonoscopy and upper GI endoscopy** | Indication: chronic ileocaecal changes on prior imaging. **Ileocolonoscopy: three small terminal-ileal ulcers with normal intervening mucosa; biopsy subsequently showed mild active chronic ileitis, reported as nonspecific.** Histology was still pending at this date. One rectal polyp removed. Upper GI endoscopy: rapid urease (CLO) test positive. **No biliary instrumentation, no sphincterotomy, no antibiotic prophylaxis.** | Histology pending at this date. No change to biliary management. |
| 16 Feb 2026 | Day 0 | **Acute presentation — emergency admission** | Two-day history of fever, progressive RUQ and epigastric pain, nausea and jaundice. **No antimicrobial exposure of any kind before admission.** Vitals: T 38.9 °C; HR 108/min; BP 118/74 mmHg (MAP 89); SpO₂ 98% room air; GCS 15. Scleral icterus; RUQ and epigastric tenderness; Murphy’s sign not elicitable (post-cholecystectomy).  **TG18 diagnosis — definite acute cholangitis (A + B + C):** A fever 38.9 °C with WBC 14.2 × 10³/µL and CRP >100 mg/L; B jaundice with total bilirubin 26 mg/dL and abnormal liver biochemistry; C mild intrahepatic ductal dilatation with distal CBD contents.  **TG18 severity — Grade II, 2 of 5 criteria met:** WBC 14.2 × 10³/µL (>12) MET; total bilirubin 26 mg/dL (≥5) MET; temperature 38.9 °C (≥39 °C) NOT met; age 42 years (≥75) NOT met; albumin 3.4 g/dL (threshold 2.45 g/dL = 0.7 × lower reference limit 3.5) NOT met.  **Grade III:** no organ dysfunction identified on the recorded variables — MAP 89 mmHg with no vasopressor; GCS 15; creatinine 1.1 mg/dL (97 µmol/L; threshold >2.0 mg/dL); INR 1.3 (threshold >1.5); platelets 228 × 10³/µL (threshold <100). **Not formally assessable:** the respiratory criterion (PaO₂/FiO₂ <300) — arterial blood gas analysis was not performed and only SpO₂ 98% on room air is recorded; and the oliguria component — quantitative urine output is not available in the record.  Biochemistry: ALT 1020 (18.5 × ULN); AST 564; GGT 312; ALP 298 U/L; total bilirubin 26 mg/dL (445 µmol/L); direct bilirubin 18 mg/dL; albumin 3.4 g/dL (34 g/L); INR 1.3; platelets 228 × 10³/µL; CRP >100 mg/L; WBC 14.2 × 10³/µL; amylase 4830 U/L (38.6 × ULN); lipase 680 U/L.  Alternative-cause screen: HBsAg, anti-HCV and anti-HAV IgM negative; HEV serology not performed. Medication review: no recent new drugs, herbal remedies or supplements; alcohol use denied.  Triphasic CT: mild intrahepatic ductal dilatation; distal CBD 3–4 hyperattenuating filling defects (greatest ≈4 mm); mild periductal fat stranding; **no dominant calculus**; Balthazar Grade A; clips in expected position, no Mirizzi morphology. | Two sets of blood cultures sent **before** the first antibiotic dose. IV piperacillin–tazobactam **4.5 g every 8 hours (q8h; 13.5 g/day)** commenced, with IV fluid resuscitation, fasting and analgesia. Metformin withheld; basal–bolus insulin started (glargine 18 U nocte; lispro 4 U three times daily pre-meal).  **TG18 Grade II severity established the indication for early biliary drainage at presentation.** Immediate ERCP was not feasible because of endoscopy-suite and anaesthetic availability. |
| 17 Feb 2026 | Day 1 | **24-hour severity reassessment** | Fever persisting: T 38.5 °C; HR 104/min; BP 122/76 mmHg; SpO₂ 98%; GCS 15. Jaundice and tenderness unchanged. Severity reassessed: Grade II maintained; no Grade III organ dysfunction identified on the recorded variables. | IV piperacillin–tazobactam continued at 4.5 g q8h; fluid resuscitation maintained; serial clinical and biochemical monitoring. Drainage indication unchanged and still in force. |
| 18 Feb 2026 | Day 2  (pre-ERCP) | **48-hour reassessment during the unavoidable interval** | Fever 38.3 °C persisting; RUQ and epigastric pain persisting; jaundice persisted (total bilirubin 24 mg/dL, 410 µmol/L).  Biochemistry: ALT 837; AST 384; GGT 278; ALP 261 U/L; direct bilirubin 17 mg/dL; albumin 3.3 g/dL (33 g/L); **CRP 86 mg/L**; **WBC 12.8 × 10³/µL**; INR 1.2; platelets 215 × 10³/µL; creatinine 1.0 mg/dL; glucose 196 mg/dL (10.9 mmol/L); amylase 501; lipase 420 U/L.  **These persistent clinical and biochemical abnormalities reinforced the urgency of drainage during the unavoidable delay. They did not constitute a separate TG18 severity criterion and did not create the drainage indication, which Grade II severity had already established on Day 0.**  MRCP not performed: the drainage indication was already established and further cross-sectional imaging could not have altered it or expedited source control. | IV antimicrobial and supportive therapy continued. ERCP scheduled for the earliest available endoscopy-suite slot. |
| 19 Feb 2026 | Day 3  (ERCP) | **ERCP at the earliest available opportunity — biliary drainage** | Oedematous ampulla; wire-guided CBD cannulation; sphincterotomy.  **Macroscopic purulent bile identified at ductal entry** — direct intra-procedural evidence of suppurative biliary infection; this is not itself a TG18 diagnostic or severity criterion. Fluoroscopy: 3–4 filling defects in the distal CBD (greatest ≈4 mm); **no dominant calculus**.  Three balloon sweeps: copious biliary sludge and purulent material extracted. Repeat fluoroscopy confirmed CBD clearance (pre-clearance 8 mm; post-clearance 7 mm). Pancreatic duct not cannulated; no stent placed. Bile aspirate sent for culture after 48 hours of antimicrobial therapy; it was not submitted for crystal microscopy or chemical analysis.  The bile culture subsequently showed no growth at five days.  A documented search of the institutional PACS and endoscopy unit records did not retrieve the original procedural fluoroscopic and endoscopic image files. | IV antimicrobial therapy continued after the procedure. Clinical monitoring continued. |
| 20 Feb 2026 | Day 4 | **Post-ERCP Day 1 — early recovery** | Fever resolved: T 37.1 °C. HR 92/min; BP 124/78 mmHg; SpO₂ 98%; GCS 15. Abdominal tenderness markedly diminished; jaundice clinically improving; tolerating oral fluids. Blood cultures (Day 0, at 48 h): no growth. Glucose 158 mg/dL (8.8 mmol/L) under insulin. | Diet reintroduced. IV antimicrobial therapy continued. Clinical monitoring ongoing. |
| 21 Feb 2026 | Day 5 | **Marked biochemical improvement** | Total bilirubin 14 mg/dL (239 µmol/L; −46% from peak); direct bilirubin 9 mg/dL; ALT 521; AST 210; GGT 180; ALP 194 U/L; albumin 3.6 g/dL; CRP 38 mg/L; WBC 9.4 × 10³/µL; platelets 236 × 10³/µL; creatinine 0.9 mg/dL; glucose 138 mg/dL (7.7 mmol/L).  Amylase 142 U/L (−97% from peak, still above the ULN of 125); lipase 190 U/L.  Blood cultures (drawn Day 0, before any antimicrobial): final report at five days — no growth. Preceding antimicrobial exposure may have reduced the yield of the bile culture obtained on Day 3; neither result excludes biliary infection. | Continued clinical and biochemical monitoring. |
| 22 Feb 2026 | Day 6 | **Discharge** | Clinically well; afebrile; tolerating a normal diet; jaundice resolving. | Discharged. Outpatient gastroenterology and endocrinology follow-up arranged. |
| 23–24 Feb 2026 | Days 7–8 | **Early post-discharge outpatient review** | ALT 189 U/L; AST 74; GGT 89; ALP 130 U/L; total bilirubin 2.1 mg/dL (36 µmol/L); direct bilirubin 1.1 mg/dL; albumin 3.9 g/dL; INR 1.0; platelets 252 × 10³/µL; amylase 88; lipase 95 U/L (above the ULN of 78); CRP 9 mg/L; WBC 7.2 × 10³/µL; creatinine 0.9 mg/dL; glucose 112 mg/dL (6.2 mmol/L). | Outpatient gastroenterology and endocrinology follow-up continued. |
| 16 May 2026 | Month 3 | **Three-month follow-up — documented recovery** | **Hepatobiliary, inflammatory, haematological and renal parameters had normalised:** ALT 38; AST 29; GGT 41; ALP 102 U/L; total bilirubin 0.9 mg/dL (15 µmol/L); direct bilirubin 0.3 mg/dL; albumin 4.1 g/dL; INR 1.0; platelets 268 × 10³/µL; CRP <5 mg/L; **WBC 6.8 × 10³/µL**; creatinine 0.9 mg/dL; fasting glucose 97 mg/dL (5.4 mmol/L).  **HbA1c improved from 9.7% to 8.3% and therefore remained abnormal.**  No recurrent biliary symptoms, jaundice or fever. | Recovery documented. Gastroenterology and endocrinology follow-up continuing. |

***Laboratory reference intervals (Palestine Medical Complex, Ramallah):*** *ALT 0–55 U/L; AST 5–34 U/L; GGT 12–64 U/L; ALP 40–150 U/L; total bilirubin 0.2–1.2 mg/dL; direct bilirubin 0.0–0.5 mg/dL; albumin 3.5–5.0 g/dL; platelets 150–400 × 10³/µL; amylase 25–125 U/L; lipase 8–78 U/L; CRP <5 mg/L; WBC 4.0–11.0 × 10³/µL; fasting glucose 70–100 mg/dL; creatinine 0.7–1.3 mg/dL. A separate reference interval for INR is not issued by the reporting laboratory. SI conversion: bilirubin (mg/dL) × 17.1 = µmol/L; glucose (mg/dL) × 0.0555 = mmol/L; creatinine (mg/dL) × 88.4 = µmol/L; albumin (g/dL) × 10 = g/L; ×10³/µL is numerically identical to ×10⁹/L.*

***Abbreviations:*** *ALP, alkaline phosphatase; ALT, alanine aminotransferase; AST, aspartate aminotransferase; CBD, common bile duct; CLO, Campylobacter-like organism; CRP, C-reactive protein; ERCP, endoscopic retrograde cholangiopancreatography; GCS, Glasgow Coma Scale; GGT, gamma-glutamyl transferase; HAV/HBV/HCV/HEV, hepatitis A/B/C/E virus; HbA1c, glycated haemoglobin A1c; HR, heart rate; INR, international normalised ratio; MAP, mean arterial pressure; MRCP, magnetic resonance cholangiopancreatography; PACS, picture archiving and communication system; RUQ, right upper quadrant; SpO₂, peripheral oxygen saturation; T, temperature; TG18, Tokyo Guidelines 2018; ULN, upper limit of normal; WBC, white blood cell count.*
